# Derivation of *healthy* hepatocyte-like cells from a female patient with ornithine transcarbamylase deficiency through X-inactivation selection

**DOI:** 10.1038/s41598-022-06184-w

**Published:** 2022-02-10

**Authors:** Ramon Santamaria, Maria Ballester, Guillem Garcia-Llorens, Francisco Martinez, Marina Blazquez, Carmen Ribes-Koninckx, Jose V. Castell, Torsten Wuestefeld, Roque Bort

**Affiliations:** 1 Experimental Hepatology Unit, Instituto de Investigación Sanitaria La Fe, CIBERehd, Hospital Universitari i Politècnic La Fe, Avda. Fernando Abril Martorell 106, 46026 Valencia, Spain; 2 Genetics Unit, Instituto de Investigación Sanitaria La Fe, Hospital Universitari i Politècnic La Fe, 46026 Valencia, Spain; 3 Coeliac Disease and Inmunopathology Research Unit, Instituto de Investigación Sanitaria La Fe, Pediatric Gastroenterology, Hospital Universitari i Politècnic La Fe, 46026 Valencia, Spain; 4 Biochemistry and Molecular Biology Department, Universidad de Valencia, Valencia, Spain; 5 Laboratory for In Vivo Genetics & Gene Therapy, Genome Institute of Singapore, A*STAR & National Cancer Centre Singapore, School of Biological Science, SingHealth & Adj. Ass.-Prof. Nanyang Technological University, 60 Biopolis Street, #02-01 Genome, Singapore, 138672 Singapore

**Keywords:** Mechanisms of disease, Metabolic disorders, Molecular medicine

## Abstract

Autologous cell replacement therapy for inherited metabolic disorders requires the correction of the underlying genetic mutation in patient’s cells. An unexplored alternative for females affected from X-linked diseases is the clonal selection of cells randomly silencing the X-chromosome containing the mutant allele, without in vivo or ex vivo genome editing. In this report, we have isolated dermal fibroblasts from a female patient affected of ornithine transcarbamylase deficiency and obtained clones based on inactivation status of either maternally or paternally inherited X chromosome, followed by differentiation to hepatocytes. Hepatocyte-like cells derived from these clones display indistinct features characteristic of hepatocytes, but express either the mutant or wild type OTC allele depending on X-inactivation pattern. When clonally derived hepatocyte-like cells were transplanted into FRG^®^ KO mice, they were able to colonize the liver and recapitulate OTC-dependent phenotype conditioned by X-chromosome inactivation pattern. This approach opens new strategies for cell therapy of X-linked metabolic diseases and experimental in vitro models for drug development for such diseases.

## Introduction

Ornithine transcarbamylase (OTC; EC 2.1.3.3) deficiency (OTCD; OMIM#311250) is an X-linked (Xp21.1)^1^ rare disease with a prevalence of 1 in 40,000–80,000^2–6^ caused by an inborn error of metabolism of the urea cycle. Patients are classified as neonatal onset (perinatal detection, severe symptoms and high mortality rate) and late-onset (non-neonatal, very variable symptoms and lower mortality rate). A total of 417 disease-causing mutations in the OTC gene were compiled in the last update^7^. The mutation included in this report consisting of an inframe de novo mutation of either E272 or E273, was first reported^8^ in a male presenting with hyperammonemic coma at an age of 10 months, who expressed only 5% OTC activity in the liver, and who did well with mild protein restriction (30 g protein/day). His mother and sister were clinically asymptomatic carriers. Another male hemizygous for this mutation was very recently reported^9^. The child presented hyperammonemic coma at 7 years old, with decreased plasma citrulline and increased urinary orotate, with rapid recovery following hemodialysis, without new bouts of hyperammonemia in the subsequent four years, without treatment. Three female carriers in the same family were asymptomatic as well as a hemizygous male cousin (he had normal plasma ammonia and blood amino acids). Thus, both unrelated male patients reveal partial, mild deficiency, linked to mutations in exon 8 of OTC gene, which affect the SMG loop of the OTC protein, a loop important for the opening and closing of the active site without being essential for catalysis or for folding of the protein core of the OTC subunit^10,11^.

OTCD paradoxically has a significant variable incidence among heterozygous females compared to other X-linked diseases (Table S1). Some diseases not included in the table, such as in Fabry disease, present high prevalence of symptomatic heterozygous females, but it is due to the accumulation of neutral glycosphingolipids caused by a deficient alpha-galactosidase A; in fact, X-inactivation is random in heterozygous females of Fabry disease (Maier et al., 2006). Unbalanced or skewed X-chromosome inactivation (XCI) is currently recognized as a possible cause for clinically relevant X-linked recessive disorders in heterozygous females^12,13^. An alternative hypothesis without X-skewing is an unfavorable lionization where cells expressing the mutant allele reside in the periportal area compared with a situation where mutant hepatocytes reside in the pericentral area; this is the consequence of hepatic zonation of OTC expression and urea cycle activity. The symptoms in heterozygous OTCD females is variable, with episodes of headaches, psychotic episodes, confusion, erratic behavior, non-specific gastrointestinal upset, recurrent vomiting, and protein avoidance, prone to misdiagnosis^14,15^. Current US and European consensus statements recommend to increase monitoring of these patients^15^.

The process of X-inactivation gives rise to a random transcriptional silencing of one of the two X chromosomes present in each female cell of the epiblast during early embryonic development^16^. Once X-inactivation is initiated, inactivated X chromosome remains silent for the rest of that cell’s life and the X-inactivation pattern is passed on to its daughter cells^17^. In the general population, the X-inactivation ratio shows a Normal distribution with the mean around 50%, while extreme skewing (> 90%) was estimated to be present in 3.6% of female unaffected blood cells^18^. Most of the skewing observed in humans are not the consequence of an inherited tendency to inactivate a particular X chromosome^19^. Making use of this phenomenon, in the present paper we have explored the feasibility of selecting fibroblast clones from a heterozygous OTC female, having spontaneously silenced one of the X chromosomes (either the paternal or the maternal), differentiated to hepatocytes phenotypically expressing the wild type or the deficient OTC gene, and recapitulating OTCD in mice transplanted with the mutant cells.

## Results

### Isolation of clonal cell populations expressing either maternally or paternally inherited X chromosome from a heterozygous OTC female patient

**Figure 1:**
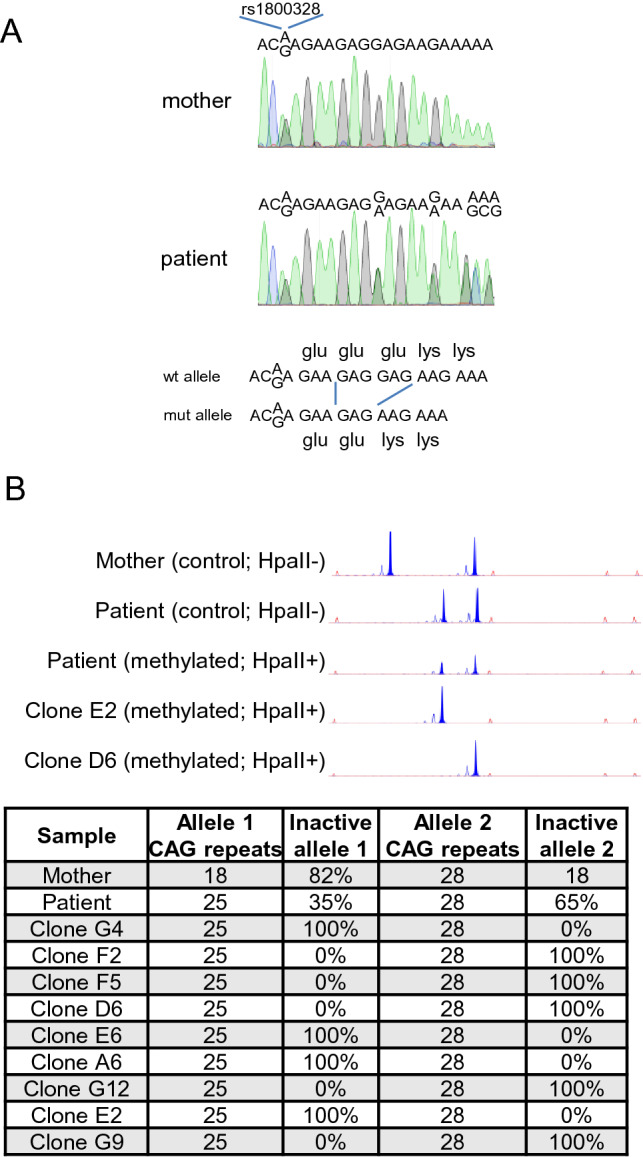
Derivation of clonal populations of HDF expressing either maternally or paternally inherited X chromosome from a female heterozygous patient of OTCD. (**A**) Biallelic genomic sequence corresponding to a region of exon 8 of the OTC gene isolated from the mother and the patient. Mother’s and patient’s region contains SNP rs1800328 in heterozygosity. Patient´s sequence is compatible with heterozygous GAG deletion in position 814–816 or 817–819. (**B**) X chromosome inactivation in genomic DNA samples from mother´s buccal mucosa, patient fibroblasts and 9 clonal populations.

We isolated dermal fibroblasts from a skin punch biopsy from a female patient of OTCD. Sequencing of genomic DNA was compatible with heterozygosity for a GAG deletion in position 814–816 (NM_000531.5:c.814_816del) or 817–819 (NM_000531.5:c.817_819del) resulting in the deletion of one amino acid of the OTC protein (p.Glu272del or p.Glu273del), which otherwise preserves the integrity of the reading frame (Fig. 1A). This mutation was previously reported^10^, and the in-frame deletion has been additionally reported in two unrelated male patients^8,9^. In close proximity, we found the polymorphism rs1800328, resulting in the missense variant p.Gln270Arg, a variant present in 4% of X chromosomes in the European population (gnomAD).

X-inactivation ratios were assessed by DNA methylation status at the AR *locus* in genomic DNA isolated from the patient and her mother. XCI analysis was informative to discriminate both, maternally (28 CAG repeats) and paternally (25 CAG repeats) inherited X chromosome from the patient (Fig. 1B). Fibroblasts were expanded and cloned by limiting dilution to prepare homogeneous populations that express either maternally or paternally inherited X chromosome. XCI in the patient fibroblasts was not skewed (35:65), indicating a non-inherited defective XCI. Four (G4, E6, A6 and E2) and five (F2, F5, D6, G12 and G9) out of ten clones had fully silenced either paternally or maternally inherited X chromosome.

### Direct reprogramming of clonal fibroblasts to induced hepatocyte-like cells (iHEP)

iHEP derived from human dermal fibroblasts (HDF) by direct reprogramming are proliferation arrested, precluding functional characterization in animal models. To override such limitation, we first generated immortalized HDF using a lentivirus-mediated expression of SV40 large T antigen^20^. Two clonal HDF populations expressing either maternally or paternally inherited X chromosome were immortalized i.e. HDF^LT^-E2, HDF^LT^-E6 (maternal) and HDF^LT^-D6, HDF^LT^-F5 (paternal) and subsequently reprogrammed to iHEP^LT^^21^. iHEP^LT^ phenotype was strongly dependent on cell density (Supplementary Fig. S1). Thus, seeding protocol was optimized as follows, cells were seeded in collagenized plates at 54,000 cells/cm^2^ in HMM media and cultured for 6 days before the analysis.

**Figure 2:**
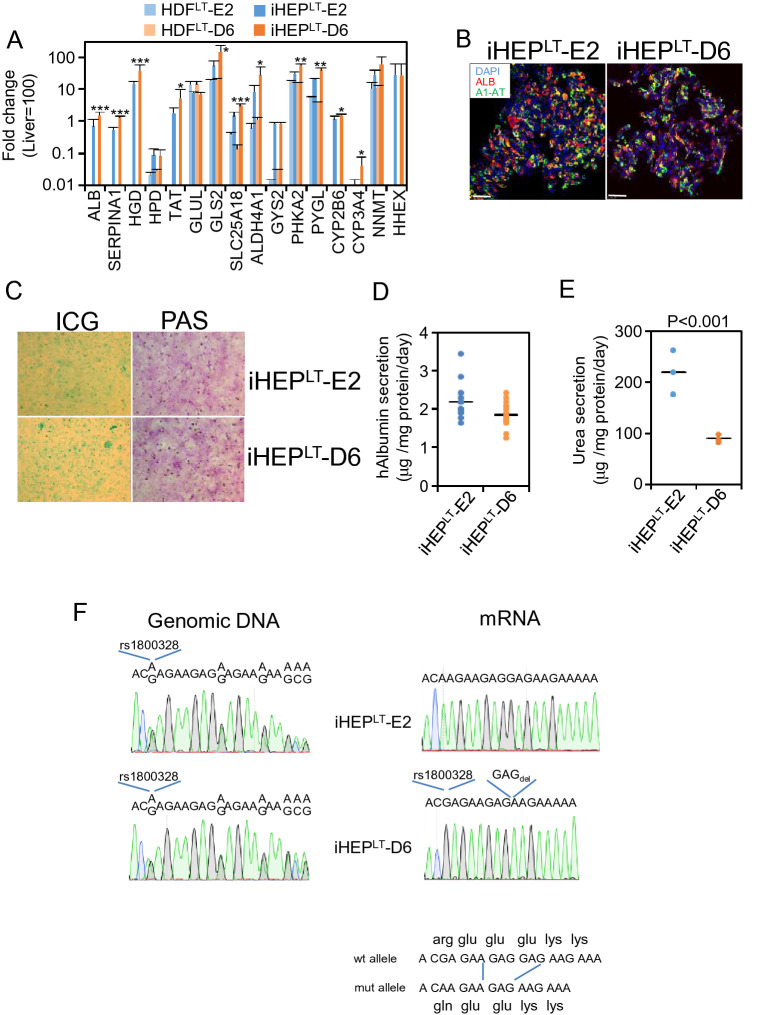
Characterization of iHEP^LT^ in vitro. (**A**) mRNA level of multiple hepatic genes was quantified by qRT-PCR, normalized to PBGD and expressed relative to levels in human liver. *, *p* < 0.05; **, *p* < 0.01; ***, *p* < 0.005; (n = 5 per group). HDF^LT^ were infected with an insert-less lentiviral vector as control. (**B**) Representative fluorescence images of iHEP^LT^-E2 and iHEP^LT^-D6 immunostained with antibodies against human albumin and α1-antitrypsin. Nuclei were stained with DAPI. Bar equals 25 µm. (**C**) Indocyanine green uptake and PAS staining. (**D**) Human albumin contained in 24-h cell media quantified by ELISA (n = 13). (**E**) Urea secretion into cell media after 24 h incubation in NH_4_Cl 2 mM (n = 3 per group). (**F**) Sequencing of genomic DNA and retrotranscribed *OTC* mRNA corresponding to the region of exon 8 containing Glu deletion. Genomic DNA and mRNA was obtained from cultured iHEP^LT^. Mutant allele (expressed in clone D6) contains also SNP rs1800328.

We compared the expression of a series of genes encoding secreted proteins (ALB and SERPINA1), enzymes of tyrosine metabolism (TAT, HPD and HGD), enzymes involved in glutamine/glutamate metabolism (GLS2, GLUL and ALDH4A1), mitochondrial glutamine transporter (SLC25A18), enzymes involved in glycogen metabolism (GYS2, PHKA2 and PYGL), enzymes involved in xenobiotic metabolism (CYP2B6, CYP3A4 and NNMT) and an embryonic liver marker (HHEX) before (HDF^LT^-E2 and HDF^LT^-E6) and after cell reprogramming (iHEP^LT^-E2 and iHEP^LT^-D6) (Fig. 2A). Reprogrammed cells activated multiple hepatocyte markers, except GLUL which is down-regulated as expected^22^. However, genes characteristic of a fully differentiated hepatocyte such as HPD or CYP3A4, were not within the range of the human liver. In conclusion, our iHEP^LT^ do not entirely recapitulate mature liver function. Of note, we found statistically significant difference in selected genes between iHEP^LT^ populations expressing either maternally or paternally inherited X chromosome. Analysis of two additional clones, one per clonal type i.e. iHEP^LT^-E6 and iHEP^LT^-F5, partially confirmed these differences (Fig. S2). Nevertheless, differences between clones within a clonal type (maternal or paternal X chromosome inherited) did not show a clear advantage, thus we continued the investigation with clones iHEP^LT^-E2 and iHEP^LT^-D6.

Functional analysis indicated that iHEP^LT^-E2 and iHEP^LT^-D6 synthetized significant amounts of albumin and α1AT, transported ICG and accumulated glycogen (Fig. 2B,C). They secreted high amounts of albumin to cell media and produced urea in vitro (Fig. 2D,E). In fact, albumin secretion was in the range of primary cultured human hepatocytes (1.42 ± 0.12 μg/mg protein/day^23^). As expected, urea production was significantly higher in iHEP^LT^-E2 compared to iHEP^LT^-D6. Forward and reverse genomic sequencing confirmed the heterozygosity for a GAG deletion in either position 814–816 or 817–819 as well as the presence of polymorphism rs1800328 (p.Gln270Arg). mRNA sequencing after retrotranscription to cDNA indicated that iHEP^LT^-E2 expresses the wt OTC allele, thus having silenced the X-chromosome containing the mutant OTC allele, while iHEP^LT^-D6 expresses the mutant OTC allele (Fig. 2F). Interestingly, the mutation is part of the G(R/Q)EEEKKK motif, in cis with p.Gln270Arg (rs1800328), only 9 bases upstream of CAG deletion. In conclusion, it was feasible to derive hepatocyte-like cells from an OTCD female patient that selectively express OTC wt allele and thus can be considered as a “healthy” cell.

### In vivo transplantation of iHEP^LT^-E2 and iHEP^LT^-D6

The generation of large numbers of iHEP^LT^ allowed us to assess the functionality of iHEP^LT^-E2 and iHEP^LT^-D6 in vivo. We designed a transplantation experiment into spf-ash mice, a spontaneous mutant mice expressing a hypomorphic OTC, widely used as a model for human OTC deficiency^24^. On a normal chow diet (18% protein) spf-ash mice develop orotic aciduria without any sign of hyperammonemia (^25^ and Supplementary Fig. S3). In fact urinary orotic acid is a surrogate marker for OTCD in gene therapy in humans and spf-ash mice^26–28^.

iHEP^LT^-E2 and iHEP^LT^-D6 were transplanted into spf-ash mice. It is important to remark that spf-ash mice are immunocompetent and fully viable under normal chow diet; thus, animals were subjected to an immunosuppression protocol with tacrolimus prior to human cells transplantation. Mice were also given an over dosage of 300 mg/kg acetaminophen (APAP) intraperitoneally to induce liver damage and facilitate engraftment of human cells. However, interim determination of human albumin in sera two weeks after transplantation below 2 ng/mL forced us to discontinue further studies.

**Figure 3:**
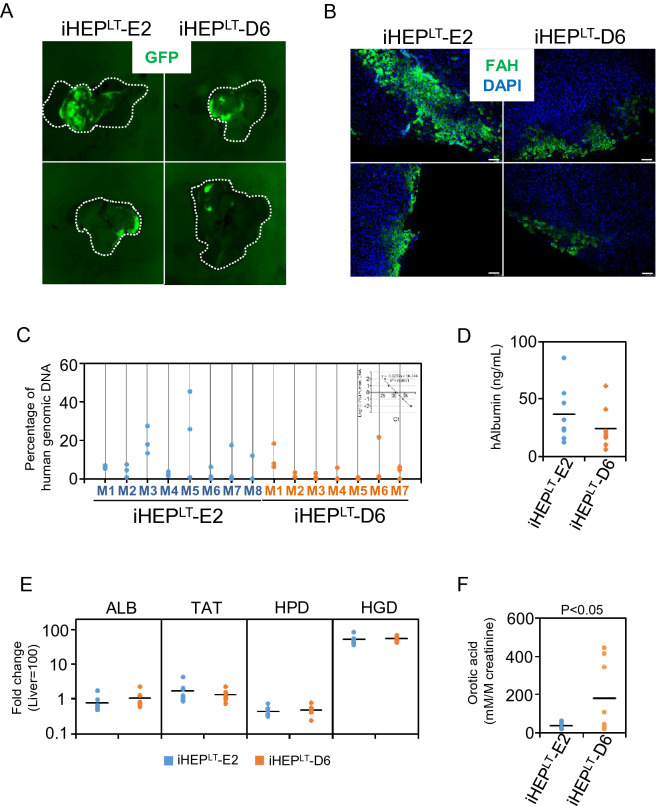
Engraftment of iHEP^LT^-E2 and iHEP^LT^-D6 and mutant phenotype recapitulation of iHEP^LT^-D6 in vivo. (**A**) Representative whole liver fluorescence images of iHEP^LT^-E2 and iHEP^LT^-D6 in livers extracted from FRG KO mice. iHEP^LT^ are fluorescent due to GFP expression from the FOXA3-eGFP lentiviral vector. Images correspond to M3 and M5 for iHEP^LT^-E2 and M1 and M6 for iHEP^LT^-D6 from Figure (**C**). (**B**) The presence of iHEP^LT^-E2 and iHEP^LT^-D6 in the liver parenchyma of transplanted FRG KO mice was confirmed by immnostaining with antibodies against FAH. Nuclei were stained with DAPI. Bar equals 50 µm. (**C**) Percentage of human genomic DNA contained in pieces of liver from transplanted FRG KO mice. We isolated genomic DNA from three pieces of the liver and quantify human genomic OTC and PBGD by qPCR. (**D**) Human albumin present in sera from transplanted FRG KO mice determined by ELISA. (**E**) mRNA level of several hepatic genes was quantified by qRT-PCR and expressed relative to levels in human liver. Only pieces expressing GFP by qRT-PCR were included in the analysis (n = 7 per group). (**F**) Orotic acid in urine obtained from transplanted FRG KO mice.

To overcome the low engraftment in spf-ash mice and still characterize OTC functionality of our cells in vivo, we switched to the FRG^®^ KO mice^29^ a gold-standard model for liver engraftment. Whole organ fluorescent pictures revealed a significant cell engraftment in areas proximal to the infusion point in the liver (Fig. 3A). Several pieces of the liver close to the fluorescent domain were prepared and processed for FAH immunohistochemistry and genomic DNA extraction. FAH-positive patches of human cells could be found in sections of the liver extracted from transplanted mice (Fig. 3B). Engraftment of iHEP^LT^ in the host liver was calculated by genomic qPCR analysis for human-specific OTC and PBGD gene sequences (Table S2). The percentage of engraftment was highly variable intra and inter mice (Fig. 3C). Although iHEP^LT^-E2 seemed to have better engraftment capacity than iHEP^LT^-D6, the difference did not reach statistical significance (*p* > 0.09). Consistent with this result, human albumin level was slightly higher in sera of mice transplanted with iHEP^LT^-E2 compared to iHEP^LT^-D6 (Fig. 3D) without reaching statistical significance. Regarding further maturation of iHEP cells within the liver environment, mRNA expression of HPD and HGD increased significantly (compare with Fig. 2A), while ALB and TAT did not change (Fig. 3E). Finally, mice transplanted with OTC-defective iHEP^LT^-D6, excreted in urine more orotic acid than those transplanted with iHEP^LT^-E2, expressing the wt allele (Fig. 3F). Considering that both iHEP^LT^ clones colonized the host liver equally, we confirmed that iHEP^LT^-D6 recapitulate the mutant OTC phenotype while iHEP^LT^-E2 displays a normal OTC function.

## Discussion

In the present study, we aimed to obtain OTC-functional hepatocyte-like cells upon cloning and direct reprogramming of dermal fibroblasts isolated from an OTCD heterozygous female patient. We utilized fibroblasts isolated from a late-onset patient (patient 23 in^10^) with a Glu deletion in position 272 or 273 within the G(R/Q)EEEKKK motif. The patient presented pronounced hepatopathy at 5 months of age followed by vomiting and lethargy at 15 months of age upon animal protein intake, with abnormal citrulline, glutamine and ammonia levels requiring, for normalization, chronic mild protein restriction, arginine and phenylbutyrate. This, and the strong production of orotate by this patient’s dermal fibroblasts-derived iHEP that only express the mutant allele, suggest a drastic effect of the mutation, resulting in quite important blockage of the utilization of carbamoyl phosphate, which, by accumulating in large amounts^30^, outflows into the route of pyrimidines biosynthesis, yielding orotate. Disease manifestations in female carriers of OTCD are attributed to skewed X-inactivation in the liver^12,13,31^. Ethical concerns precluded us from determining the degree of X-inactivation skewing in the liver of the patient. Nevertheless, we did not find skewed X silencing in primary cultured fibroblasts from the skin of the patient, excluding an inherited tendency to inactivate a particular X chromosome.

The coexistence in the mutant allele of this female patient of an infrequent OTC sequence variant, p.Gln270Arg, just three residues upstream of the deleted glutamate, was revealed by mRNA sequencing and could have increased the deleterious effects of the Glu deletion. The p.Gln270Arg OTC variant, recorded with about 4% frequency in the gnomAD database is considered a trivial polymorfism that has been recorded in gnomAD in 62 homozygotes and 2268 heterozygotes. Unhappily, in the reported structures of human OTC only the rare Arg270 variant of human OTC was studied (see for example^32^), and thus, no information exists about the structural differences with the much more frequent Gln270 form. However, in the human OTC structure (Supplementary Fig. S4) the side-chain of Arg270 makes a salt bridge with the side-chain carboxylate group of glutamate 273. As Glu273 is the third glutamate of three correlative glutamic acid residues, the loss of one glutamate is likely to result in aberrant interaction of the arginine side-chain with the side chain of Glu272, locking the SMG loop in a fixed conformation, drastically curtailing the opening and closing of the entry to the enzyme active site. In contrast, in the more frequent Gln270 form, the weaker interaction of the non-ionic hydrogen bonding of the amidic group of Gln270 with the O atom of the side-chain carboxylate of glutamate 272 in the mutant OTC with only Glu 272 or 273 deletion is unlikely to lock so firmly the mutant loop, possibly allowing better functionality and lesser hampering of OTC function.

To prove in vivo the functionality of the iHEP^LT^ expressing the wild type allele of OTC, we attempted to rescue spf-ash mice by transplantation of iHEP^LT^-E2. However, low levels of human albumin in mice sera suggested low engraftment and precluded further analysis. Low engraftment of iHEP transplantation in wt mice using APAP insult was reported recently^33^. We confirmed severe necrosis in the liver after APAP treatment (300 mg/kg) by H&E staining (data not shown). Still, we cannot exclude an immunosuppression failure, although tacrolimus at 1–3 mg/Kg/day is an extended dosage used in mice immunosuppression^34^. This result evidences the current limitations to achieve a high degree of engraftment in animal models, when there is not a selective advantage for infused cells to overcome resident hepatocytes. As an alternative we used FRG^®^ KO mice, a mice model par excellence for liver engraftment experiments^29^. Removal of NTBC treatment in these animals causes autologous hepatocyte cytotoxicity largely facilitating the engraftment of infused cells. Since, FRG^®^ KO mice are not OTC deficient and do not present elevated orotic acid levels in urine, we had to reverse the rationale of the experiment by transplanting in parallel iHEP^LT^-D6, the clone expressing the mutant OTC allele. Consistent with this approach, urinary orotic acid, was significantly higher in mice transplanted with OTC-defective iHEP^LT^-D6 than in those transplanted with iHEP^LT^-E2, expressing wt allele.

Hepatocyte cell therapy emerged several years ago as a possible approach to treat liver-originated metabolic disorders (Crigger-Najjar, urea cycle defects…)^35,36^. Principal bottlenecks associated with liver cell therapy are off-target genome editing in mutant cells, safety of cells after manipulation with viral vectors, and limited engraftment of cells in the host liver. Our strategy provides a proof-of-concept for an alternate strategy avoiding genome edition by cell selection and expansion procedures. However, it can only be contemplated in X-linked diseases and using a protocol avoiding the use of oncogenes such as large T-antigen or lentivirus-mediated gene delivery. Similarly, several strategies have been tested to increase cell engraftment in a (near) normal liver^37–39^ that require further improvement prior to a feasible cell therapy in humans.

## Methods

### Human dermal fibroblasts

Human dermal fibroblasts (HDF) were isolated from a punch skin biopsy from a heterozygous female OTC patient. Informed consent in writing was obtained and the study protocol conformed to the ethical guidelines of the 1975 Declaration of Helsinki as reflected in approval number SAF2014-51991 by Comité Ético de la Investigación con medicamentos (CEIm; https://www.iislafe.es/es/el-iis-la-fe/organos-de-gobierno/comites-eticos/comite-etica-investigacion-medicamentos-ceim). HDF were isolated as described in detail in http://www.bu.edu/dbin/stemcells/protocols.php. Briefly, skin biopsy was incubated o/n at 37 °C in 1 mL digestion media (DMEM high glucose high glucose containing 20% fetal bovine serum, 23,500 U. Collagenase type I, 20 mg DNAse I and 1% penicillin–streptomycin). Next day, digested skin was vortexed for 20 s, centrifuged at 1500 g × 3 min and resuspended in 4 mL of incubation media (DMEM high glucose containing 20% fetal bovine serum and 1% penicillin–streptomycin). Cells were seeded in a T25 flask and left untouched for 3 days.

### Mice

B6EiC3Sn a/A-Otcspf-ash/J (spf-ash) and B6EiC3SnF1/J mice use was performed in compliance with the ARRIVE guidelines and approved by the Institutional Animal Ethics Committee of the Instituto de Investigación Sanitaria La Fe and Generalitat Valenciana (reference number 2016/VSC/PEA/00019 and 2016/VSC/PEA/00126). B6EiC3Sn a/A-Otcspf-ash/J mice (spf-ash; Stock nr 001811) and B6EiC3SnF1/J mice (wt-control breeding; Stock nr 001875) were acquired from The Jackson Laboratories and housed at the animal facilities of the Instituto de Investigación Sanitaria La Fe. Mice were bred and genotyped as recommended by Jackson Laboratories. Mice were maintained on a 12-h light/dark cycle with free access to rodent chow and water. The ages of mice subjected to transplantation experiments ranged between 8–12 weeks (adults only). Only male hemyzigous B6EiC3Sn a/A-Otcspf-ash/J mice were used in transplantation studies.

FRG^®^ KO mice use was approved by A*STAR Institutional Animal Care and Use Committee (Reference number IACUC# 151054). FRG^®^ KO mice were acquired from Yecuris™. Mice were maintained on a 12-h light/dark cycle with free access to rodent chow and water containing NTBC (16 mg/L NTBC in 3% dextrose solution) and antibiotics SMX and TMP (0.64 mg/mL and 0.128 mg/mL respectively) as recommended by Yecuris™. Cell transplantation was performed in the animal facilities of the A*STAR research Institute. The ages of mice subjected to transplantation experiments ranged between 6–12 weeks (adults only).

### Cell culture and lentivirus generation

Cells were maintained at 37 °C with 5% CO_2_ and were regularly examined with an Olympus CKX41 microscope. Human Dermal Fibroblasts and HEK293T cells were cultured in DMEM high glucose containing 10% fetal bovine serum and 1% penicillin/streptomycin. Lentivirus were generated in HEK293T cells by calcium phosphate cotransfection of the corresponding pHIV vector with pPAX2 and pMD2.G in 10:7.5:5 ratio. 48-h after cotransfection, supernatants were collected and lentivirus concentrated (12x) using Lenti-X concentrator following manufacturer´s instructions (Takara). All media was purchased from Thermo Fisher Scientific.

### Cloning and generation of HDF^LT^

Primary cultured HDF were cloned by dilution cloning as described^40^. Briefly, HDF cultured cells were trypsinized and resuspended in DMEM high glucose containing 20% fetal bovine serum and 1% penicillin/streptomycin. A suspension of 3 cells per mL was obtained by 1/100 serial dilution. Two hundred microliter cell suspension per well was seeded in a 96-well plate (0.5–1 cell/well). Only wells microscopically confirmed to contain one cell were further used. Plates were kept at 37 °C, 5% CO_2_ and 2% O_2_ for 2 weeks with a media change after 1 week. Wells containing cells were expanded and stocked. X-inactivation status was assessed in expanded clones as previously described with minor modifications^41^. Clonal HDF^LT^ were generated by infecting clonal HDF with lentivirus vector pRRLsin-SV40 T-mCherry. Cells were subjected to several 1/10 passaging until Cherry-positive cells percentage was above 99.5% by flow cytometry.

### Generation of iHEP^LT^

Clonal HDF^LT^ were reprogrammed to iHEP^LT^ using a protocol adapted from^20,22^. HDF^LT^ (18 × 10^3^cells) were seeded on collagen coated plates and infected with equal amounts of 12x concentrated lentiviral vectors encoding HNF4A, HNF1A and FOXA3. Cells were kept in HFM media: DMEM/F12 supplemented with 10% Fetal bovine serum, 0.1 mM β-mercaptoethanol, 1x MEM Non-Essential Amino Acids Solution and 4 ng/mL bFGF) for 48 h and then switched to HMM media: DMEM/F12 supplemented with 0.544 mg/L ZnCl_2_, 0.75 mg/L ZnSO_4_·7H2O, 0.2 mg/L, CuSO_4_·5H_2_O, 0.025 mg/L MnSO_4_, 2 g/L Bovine serum albumin, 0.1 g/L Ornithine, 0.03 g/L Proline, 0.61 g/L Nicotinamide, 1X Insulin–transferrin–sodium selenite media supplement, 40 ng/mL TGFα, 40 ng/mL EGF, 10 μM dexamethasone and 1% fetal bovine serum). Cells were expanded until p6 and stocked (approx. 12 × 10^6^ cells distributed in 8 vials). iHEP^LT^ phenotype was heavily dependent on cell density (Supplementary Fig. S1). Thus, seeding protocol was optimized as follows: cells were seeded in a collagenized plate at 54,000 cells/cm^2^ in HMM media. Cell media was changed daily for 6 consecutive days. At this point cells were used for in vitro analysis or animal transplantation. bFGF, TGFα and EGF were purchased to Peprotech (London, UK). Cells were maintained at 37 °C with 5% CO2 and were regularly examined with an Olympus CKX41 microscope.

### qPCR, RT-qPCR and sequencing

Genomic DNA was extracted from 3 different pieces (approx. 10 mg each) of liver from transplanted FRG^**®**^ KO mice by phenol:chloroform extraction. To quantify human genomic DNA in transplanted mouse liver, we prepared a calibration curve containing a mix of human and mouse genomic DNA ranging from 1:100 to 100:0. Specific genomic regions of human PBGD and OTC genes were amplified by qPCR using LightCycler^®^ 480 SYBR Green I Master Mix (Roche Life Science). RNA was isolated from cultured cells using E.Z.N.A.^®^ Microelute TOTAL RNA kit (Omegabiotek). qRT-PCR was performed as previously described^42^. Expression values were calculated by the formula 2^∆Ct^ using PBGD for normalization and expressed relative to human liver. Primer sequences are included in Supplemental information, Table S2. PCR fragments from genomic DNA or mRNA were purified using QIAquick PCR Purification kit (Qiagen) and sequenced by Sanger using forward (gDNA) or forward and reverse primer (cDNA).

### Immunofluorescence, periodic acid Schiff (PAS) staining protocol, indocyanine green (ICG), albumin secretion and urea secretion

Immunofluorescence was performed as previously described^42^. Cells were fixed with 4% paraformaldehyde at room temperature, and then washed three times with PBS. Cells were blocked with blocking buffer (5% normal donkey serum, 0.3% Triton X-100 in PBS) for 60 min at room temperature and then incubated with primary antibodies at 4 °C overnight in 0.1× blocking buffer. The next day cells were washed three times with PBS, and then incubated with Alexa Fluor conjugated secondary antibodies at 1/500 dilution Thermo Fisher Scientific) for 45 min at room temperature in the dark. Nuclei were stained with DAPI (200 ng/mL for 15 min). Primary antibodies used were as follows: goat anti-hALB (1:500, Bethyl Laboratories, RRID: AB_67018), rabbit anti-A1AT (1:500, Cell Marque, RRID: AB_1160952). Fluorescence images were taken in Olympus FV1000 confocal mounted on an IX81 inverted microscope.

Cells were stained by periodic acid-Schiff (PAS; Sigma) following the manufacturer’s instructions. Confluent cells were incubated with fresh HMM medium supplemented with 1 mg/mL indocyanine green at 37 °C for 30 min.

To determine the presence of human albumin in cell media and mice sera, we used a human Albumin ELISA Quantitation Set (Bethyl Laboratory) according to the manufacturer´s instructions. Ureagenesis was estimated by incubating iHEP^LT^ in HMM media without phenol red containing 2 mM NH_4_Cl for 24 h and measuring urea content in cell media with Quantichrome Urea Assay Kit (Bioessays).

### In vivo transplantation and immunohistochemistry

Orthotopic transplantation of iHEP^LT^ in male hemizygous spf-ash mice was done as previously described^43^. Ten males were randomly assigned to receive iHEP^LT^-E2 or iHEP^LT^-D6 (n = 5 per group). Animals were immunosuppressed using tacrolimus 2.5 mg/Kg/day starting 2 days before surgery. Mice (6–12 weeks) were treated with 300 mg APAP/kg to induce acute liver failure 3 h prior to cell transplantation. Acute liver failure was confirmed by histological staining in parallel treated mice. Three hours after the injection of APAP, mice were anaesthetized with a sevoflurane/O2 mixture and the lower pole of the spleen was exposed. Animals received an intrasplenic injection of 2 × 10^6^ iHEP^LT^ in 150 μL infusion medium within seconds. Four weeks after infusion, mice were sacrificed under anaesthesia (sevoflurane/O_2_ mixture). Blood was collected and serum aliquots were protected from light and stored at − 80 °C until analysis.

Orthotopic transplantation of iHEP^LT^ in FRG^®^ KO mice was done as previously described^44^. Twenty males were randomly assigned to receive iHEP^LT^-E2 or iHEP^LT^-D6 (n = 10 per group). Mice (6–12 weeks old) were deeply anesthetized by either isoflurane gas or intraperitoneal injection of Ketamine (150 mg/kg) and Xylazine (10 mg/kg). The abdominal skin was disinfected with 70% ethanol and a midline abdominal skin and muscle incision (about 3 cm long) to expose the xiphoid process was performed. The left lateral lobe was identified and a 4-0 silk thread loop was placed around on the tip of the lobe. A small-volume suspension (not more than 150 uL) containing 2 million cells in PBS were injected by a 27 gauge needle into this liver lobe tip. After withdrawal of the injection needle the silk thread loop was tighten to avoid any leakage of cells into the peritoneum. The liver lobe was returned to the peritoneum. The peritoneum was closed with a 5-0 suture. The skin was closed with 4-0 sutures. After closing the abdomen, the skin surrounding the suture was disinfected with 70% ethanol and the animal was placed on a warming pad for recovery (1–2 h). When the described procedure is performed correctly, no mortality is seen in the mice (hunched position for the first 12 h post-surgery was noticed in some animals). Buprenorphine 0.05–0.1 mg/kg (SC, every 12 h) and Baytril 5–20 mg/kg (SC, twice a day) were administered to post-surgery animals. Mice were monitored during the postoperative recovery phase and over the next 3 days post-surgery. Four weeks after infusion, mice were sacrificed under anaesthesia (sevoflurane/O_2_ mixture). Two mice in the iHEP^LT^-E2 group and three in the iHEP^LT^-D6 died before the endpoint of the experiment probably due to low engraftment. Blood was collected and serum aliquots were protected from light and stored at − 80 °C until analysis. Urine was collected by transferring the mice individually into a clean metabolic box for 1 h and transferring the urine into a 1.5 mL tube.

Immunohistochemistry procedure was described in^42^. Sections of the paraffin-embedded tissues were dewaxed and rehydrated through ethanol:water series and unmasked by boiling in sodium citrate buffer (10 mM, pH 6.0, for 10 min). Immunostaining was performed as described above. Slides were mounted with fluorescence mounting media (Dako). Primary antibody used was rabbit anti-FAH (1:200, Abcam, RRID: AB_1860393). Fluorescence images were taken in Olympus FV1000 confocal mounted on an IX81 inverted microscope.

### Orotic acid quantification in urine

Urine sampling for this study was always done in the afternoon after a 3 h fasting. Mice usually get use to the manipulation necessary for urine collection, which hampers routine sampling. To overcome this limitation, urination was stimulated by moving individual mice to an empty cage of another male. Urine was diluted 1/10 with water/FA (99.8:0.2) containing 1 ppm of the internal standard Phenylalanine-d5.

Quantification of orotic acid in urine was performed using UPLC-MSMS (QqQ) 1290–6460 from Agilent. UPLC separation was performed in an Agilent 1290 Infinity II LC system equipped with a 150 mm × 2.1 mm, 4 µm particle size Synergi-Hydro C18 column (Phenomenex Inc). Autosampler and column temperatures were set at 4 °C and 253 °C, respectively. The mobile phase was water/formic acid (99.8:0.2) (A) and acetonitrile (B). Elution gradient consisted of 3% B for 0–2 min and then linear 3–95% B in 8 min, staying at 95% B for 1 min and column equilibration with 3% B for 2 min. The flow rate was set to 0.25 mL/min and injection volume of 10 µL.

MS analysis was performed using Agilent 6460 Triple Quad Mass Spectrometer equipped with an ESI source in the negative-ion node working in multiple reaction monitoring (MRM) mode. Retention time was 2.11 min and transitions 155.1 > 111 (quantitative) and 155.1 > 67.2 (qualitative). Phenylalanine-d5 was used as internal standard at 1 ppm.

## Supplementary Information

Supplementary Information.

## Data Availability

The datasets generated during and/or analysed during the current study are available from the corresponding author on reasonable request.

## Abbreviations

bFGF: Basic fibroblast growth factor
DMEM: Dulbecco’s modified Eagle’s medium
EGF: Epidermal growth factor
HDF: Human dermal fibroblasts
HFM: Human fibroblast medium
HMM: Hepatocyte maturation medium
ICG: Indocyanine green
iHEP: Hepatocyte-like cells
iHEP^LT^: T large antigen immortalized hepatocyte-like cells
MRM: Multiple reaction monitoring
MS: Mass spectrometry
OTC: Ornithine transcarbamylase
OTCD: Ornithine transcarbamylase deficiency
PAS: Periodic acid–Schiff stain
PBS: Phosphate buffered saline
SC: Subcutaneous
TGFα: Transforming growth factor α
UPLC: Ultra performance liquid chromatography
XCI: X chromosome inactivation
