# Supplementary material for: Derivation of healthy hepatocyte-like cells from a female patient with ornithine transcarbamylase deficiency through X-inactivation selection
Source: Sci Rep. 2022 Feb 10;12:2308. doi: 10.1038/s41598-022-06184-w (PMC8831560; doi:10.1038/s41598-022-06184-w)
Supplement: Supplementary file 1 — Supplementary Information. [file 41598_2022_6184_MOESM1_ESM.pdf]

Table S1. Incidence of symptomatic female carriers in selected X-linked diseases

Disease	Female (%)	Reference
OTCD-Italy	39	(Dionisi-Vici et al., 2002)
OTCD-Spain	37	(Martín-Hernández et al., 2014)
OTCD-USA	47	(Brassier et al., 2015)
OTCD-Argentina	43	(Silvera-Ruiz et al., 2019)
Duchenne Muscular Dystrophy	0.012	(Nozoe et al., 2016)
Hemophilia A	0.7	CDC's 2006 National Hemophilia Data Set
Hemophilia B	1.3	CDC's 2006 National Hemophilia Data Set
Lesch-Nyhan síndrome	0	(Dionisi-Vici et al., 2002)

Table S2. Primer sequences used in this study

Forward human *ALB* RT-qPCR<br>5'-TAAGGAGACCTGCTTTGCCG-3'	Ballester et al., 2019a
Reverse human *ALB* RT-qPCR<br>5'-AGACAGGGTGTTGGCTTTACA-3'	Ballester et al., 2019a
Forward human *HGD* RT-qPCR<br>5'-GGACCTGATGCTGACTGCTT-3'	Ballester et al., 2019a
Reverse human *HGD* RT-qPCR<br>5'-AAGTGGCTCTTGAGTGGCTC-3'	Ballester et al., 2019a
Forward human *TAT* RT-qPCR<br>5'- TCTCTGTTATGGGGCGTTGG -3'	This manuscript
Reverse human *TAT* RT-qPCR<br>5'- TGGACAGACTGCTCAGCAAC -3'	This manuscript
Forward human *GLUL* RT-qPCR<br>5'- TGGGAACTGGAATGGTGCAG -3'	This manuscript
Reverse human *GLUL* RT-qPCR<br>5'- CCAGTTAGACGTCGGGCATT -3'	This manuscript
Forward human *GLS2* RT-qPCR<br>5'- CGAAGGTTTGCCTTGTCAGC -3'	Ballester et al., 2019a
Reverse human *GLS2* RT-qPCR<br>5'- AGAGTGTGTAGGAGTCCTGGT -3'	Ballester et al., 2019a
Forward human *HHEX* RT-qPCR<br>5'- CGGACGGTGAACGACTACA -3'	Ballester et al., 2019a
Reverse human *HHEX* RT-qPCR<br>5'- TTTGACCTGTCTCTCGCTGA -3'	Ballester et al., 2019a
Forward human *HPD* RT-qPCR<br>5'- GACGGCCAAGATCAAGGTGA -3'	This manuscript
Reverse human *HPD* RT-qPCR<br>5'- TGCACCGGTTTGGTGAAGAT -3'	This manuscript
Forward human *ALDH4A1* RT-qPCR<br>5'- CTGGACGGGGTTGTGCATT -3'	Ballester et al., 2019a
Reverse human *ALDH4A1* RT-qPCR<br>5'- CCCTGCGTGAAGGCTAAGAC -3'	Ballester et al., 2019a
Forward human *SLC25A18* RT-qPCR<br>5'- CCGACTGTGCCAGGAAACTC -3'	This manuscript
Reverse human *SLC25A18* RT-qPCR<br>5'- CCTTGCCCCCTCTAGGCTAA -3'	This manuscript
Forward human *NNMT* RT-qPCR<br>5'- GACTACTCAGACCAGAACCTGC -3'	Ballester et al., 2019a

Reverse human *NNMT* RT-qPCR<br>5'- GTCACATCACACTTCAGCACCT -3'	Ballester et al., 2019a
Forward human *CYP3A4* RT-qPCR<br>5'- CAAGAAGAACAAGGACAACATAGA -3'	This manuscript
Reverse human *CYP3A4* RT-qPCR<br>5'- TTTACAAGGTTTGAAGGAGAAGTT -3'	This manuscript
Forward human *PBGD* RT-qPCR<br>5'- CGGAAGAAAACAGCCCAAAGA -3'	Ballester et al., 2019a
Reverse human *PBGD* RT-qPCR<br>5'- TGAAGCCAGGAGGAAGCACAGT -3'	Ballester et al., 2019a
Forward human *OTC* RT-PCR-seq<br>5'- AAATGATCCATTGGAAGCAGCG -3'	This manuscript
Reverse human *OTC* RT-PCR-seq<br>5'- CTTTCTGGGCAAGCAGTGTAAAA -3'	This manuscript
Forward human $OTC_{genomic}$ PCR-seq<br>5'- TGGTACCAAGCTGTTGCTGA -3'	This manuscript
Reverse human $OTC_{genomic}$ PCR-seq<br>5'- GTAACCTGGTAACCTTGGAAAGC -3'	This manuscript
Forward human $OTC_{genomic}$ qPCR<br>5'- TGAGTTGGTTTATGGGGAAAAGAGA -3'	This manuscript
Reverse human $OTC_{genomic}$ qPCR<br>5'- CAAAGCATATCATACGGTTCACCC -3'	This manuscript
Forward human $PBGD_{genomic}$ qPCR<br>5'- CCTTGATGACTGCCTTGCCT -3'	This manuscript
Reverse human $PBGD_{genomic}$ qPCR<br>5'- AAGGCTGTTGCTTGGACTTCT -3'	This manuscript

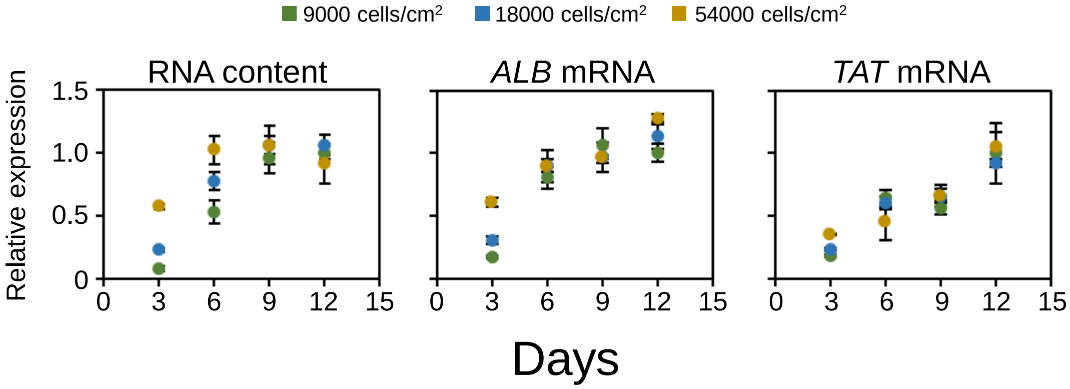

Supplementary Fig. S1. Cell density and not time in culture shapes hepatic phenotype in iHEP^{LT} cells. iHEP^{LT}-D6 were seeded at 9000, 18000 and 54000 cells/cm^2 and cultured in HMM media. Total RNA was extracted at 3, 6, 9 and 12 days. RNA content, ALB expression and TAT mRNA expression was quantified and expressed relative to the value of 9000 cells/cm^2 at 12 days (n=3).

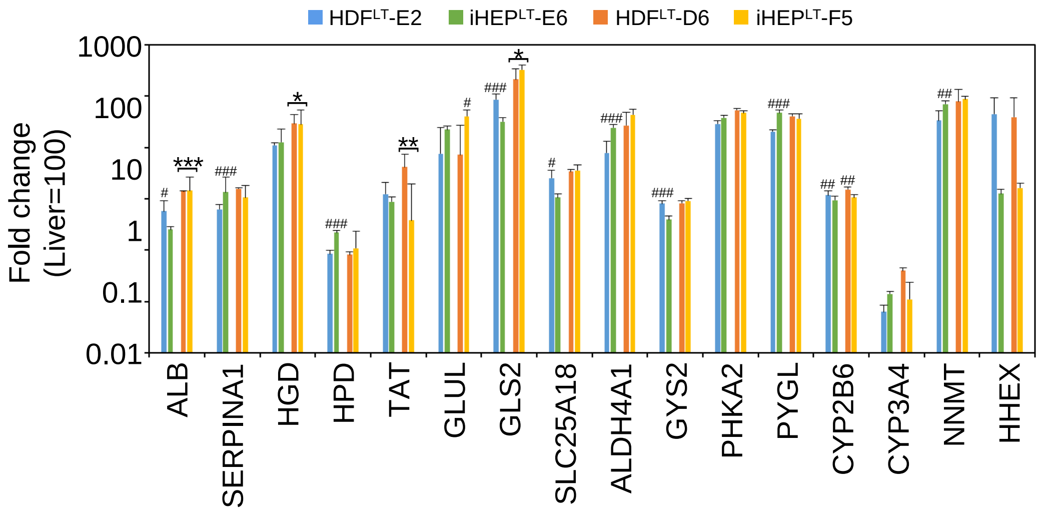

Supplementary Fig. S2. Levels of selected mRNAs, determined by qRT-PCR, in iHEP^{LT} clones expressing either maternally (iHEP^{LT}-E2, iHEP^{LT}-E6) or paternally (iHEP^{LT}-D6, iHEP^{LT}-F5) inherited X chromosome. Values are normalized with PBGD and expressed relative to levels in the human liver. Student's t results: $^{\#}$, p<0,05; $^{\#\#}$, p<0.01; $^{\#\#\#}$, p<0.005 in comparison within clonal type (iHEP^{LT}-E2 vs iHEP^{LT}-E6 or iHEP^{LT}-D6 vs iHEP^{LT}-F5); *, p<0.05; **, p<0.01 in comparison between clonal types (maternally vs paternally expressing X chromosome).

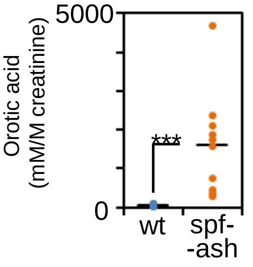

Supplementary Fig. S3. Urinary orotic acid in spf-ash mice. Orotic acid level in urine obtained from control and spf-ash mice under normal chow diet (18% protein). ***, $p<0.005$; n=9. Wt label corresponds to B6EiC3SnF1/J mice. Min-max value (wt): 33.8-100.1 mM/M creatinine; Min-max value (spf-ash): 277.8-4684.2 mM/M creatinine.

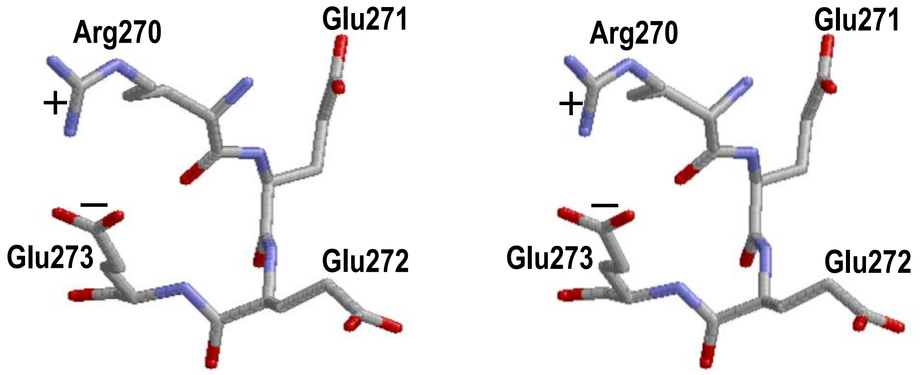

Supplementary Fig. S4. Stereo representation in sticks of the detail of the part of the SMG loop of human OTC encompassing residues 270-273. Red, blue and grey correspond to oxygen, nitrogen and carbon atoms, respectively. The positive and negative signs are shown to indicate the formation of an ion pair between Arg270 and Glu273. If Glu273 is deleted a structural shift appears possible that would allow ionic interaction between Arg270 and Glu272. No ion pairs would be formed in the more common variant Gln270. The figure corresponds to PDB file 1C9Y (Shi et al., 2000) and was drawn with PyMOL (https://pymol.org/2/).
